# One Digital Health Intervention for Monitoring Human and Animal Welfare in Smart Cities: Viewpoint and Use Case

**DOI:** 10.2196/43871

**Published:** 2023-05-19

**Authors:** Arriel Benis, Mostafa Haghi, Thomas M Deserno, Oscar Tamburis

**Affiliations:** 1 Department of Digital Medical Technologies Holon Institute of Technology Holon Israel; 2 Working Group “One Digital Health” European Federation for Medical Informatics (EFMI) Le Mont-sur-Lausanne Switzerland; 3 Working Group “One Digital Health” International Medical Informatics Association (IMIA) Chene-Bourg, Geneva Switzerland; 4 Ubiquitous Computing Laboratory Department of Computer Science HTWG Konstanz – University of Applied Sciences Konstanz Germany; 5 Peter L. Reichertz Institute for Medical Informatics of TU Braunschweig and Hannover Medical School Braunschweig Germany; 6 Working Group “Accident & Emergency Informatics” International Medical Informatics Association (IMIA) Chene-Bourg, Geneva Switzerland; 7 Institute of Biostructures and Bioimaging National Research Council Naples Italy

**Keywords:** One Health, Digital Health, One Digital Health, accident and emergency informatics, eHealth, informatics, medicine, veterinary medicine, environmental monitoring, education, patient engagement, citizen science, data science, pets, human-animal bond, intervention, ambulatory monitoring, health monitoring, Internet of Things, smart environment, mobile phone

## Abstract

Smart cities and digital public health are closely related. Managing digital transformation in urbanization and living spaces is challenging. It is critical to prioritize the emotional and physical health and well-being of humans and their animals in the dynamic and ever-changing environment they share. Human-animal bonds are continuous as they live together or share urban spaces and have a mutual impact on each other’s health as well as the surrounding environment. In addition, sensors embedded in the Internet of Things are everywhere in smart cities. They monitor events and provide appropriate responses. In this regard, accident and emergency informatics (A&EI) offers tools to identify and manage overtime hazards and disruptive events. Such manifold focuses fit with One Digital Health (ODH), which aims to transform health ecosystems with digital technology by proposing a comprehensive framework to manage data and support health-oriented policies. We showed and discussed how, by developing the concept of ODH intervention, the ODH framework can support the comprehensive monitoring and analysis of daily life events of humans and animals in technologically integrated environments such as smart homes and smart cities. We developed an ODH intervention use case in which A&EI mechanisms run in the background. The ODH framework structures the related data collection and analysis to enhance the understanding of human, animal, and environment interactions and associated outcomes. The use case looks at the daily journey of Tracy, a healthy woman aged 27 years, and her dog Mego. Using medical Internet of Things, their activities are continuously monitored and analyzed to prevent or manage any kind of health-related abnormality. We reported and commented on an ODH intervention as an example of a real-life ODH implementation. We gave the reader examples of a “how-to” analysis of Tracy and Mego’s daily life activities as part of a timely implementation of the ODH framework. For each activity, relationships to the ODH dimensions were scored, and relevant technical fields were evaluated in light of the Findable, Accessible, Interoperable, and Reusable principles. This “how-to” can be used as a template for further analyses. An ODH intervention is based on Findable, Accessible, Interoperable, and Reusable data and real-time processing for global health monitoring, emergency management, and research. The data should be collected and analyzed continuously in a spatial-temporal domain to detect changes in behavior, trends, and emergencies. The information periodically gathered should serve human, animal, and environmental health interventions by providing professionals and caregivers with inputs and “how-to's” to improve health, welfare, and risk prevention at the individual and population levels. Thus, ODH complementarily combined with A&EI is meant to enhance policies and systems and modernize emergency management.

## Introduction

### Background

The current data-driven society pushes people to seek and ask for an always-increasing number of highly personalized services [1]. Citizens of smart cities use ubiquitous and mobile technologies and expect to obtain what they need before asking for it. Thus, smart cities are models of urbanization development wherein technologies are actively deployed to enhance the quality of life (QoL) of both humans and animals [2].

Accordingly, personal data such as location, interests (eg, queries on search engines, reads on social media, answers to surveys, and purchases), contacts (eg, calls and social media connections), and activities (eg, neighborhood meetings and personal announcements) are shared continuously [3]. Concerning health-related issues, citizens, as patients, demand proactive suggestions from a modern and evolving public health sector to improve self-care and lower the number of critical events. To this end, their digitized health records encompass the entire process, from the physician’s consultation (in person or remote) to purchasing prescribed drugs (in person or on the web). People use web-based services to check their laboratory tests or reports of certified physical training. Furthermore, they may use applications for sleep monitoring that are provided by a health care management organization [4-8].

Smart cities and (digital) public health share aspects related to a healthy lifestyle. The Internet of Things (IoT; we will use *IoT* as an encompassing term including the Internet of Medical Things [IoMT] and the Internet of Animal Health Things [IoAHT]) monitors several subtopics of public health: environmental conditions, electromagnetic radiation, health conditions, fitness activities, food quality, emotions, and accidents [9-11]. Therefore, we need signals, data, and information produced by sensors in wearables or mobile apps or existing on social media networks to run health interventions [10] and cope with emergencies [12]. Accordingly, this has been called to expand the capabilities of health records. In smart cities, IoT devices also trace and track animals. Such applications reflect social and cultural norms, the safety and wellness of animals (eg, pet activity and feeding trackers [13]), and the collective composed of humans and animals [14]. In such communities, large amounts of data are produced continuously and exchanged wirelessly [15]. Its purposes range from home automation to trip management and QoL. These sensors range from microelectromechanical systems to advanced medical devices.

It is worth noting that, during the first waves of the COVID-19 pandemic, an increasing rate of shared information was observed. The need arose to timely deliver health care for both humans and animals as specific clusters of customers belonging to the same ecosystem [5,9]. Valid data collection was needed [16,17] that fit the essential elements of smart (healthy) cities [18,19]. For instance, McConnell et al [20] analyzed the influence of owning animals on stress. Other work has assessed the impact of the surrounding ecosystem on humans’ and animals’ QoL [21,22].

A practical setup for the entire data management process comprises aspects of generation, collection, transmission, storage, extraction, analysis, reporting, and decision-making as codified according to the principles of accident and emergency informatics (A&EI), with the dual purpose of preventing harm and supporting decisions [23]. Furthermore, aspects such as education, citizen engagement, and the large vision of human nature are notable elements of the One Digital Health (ODH) framework.

### The ODH Framework to Set Up an ODH Intervention

The *ODH framework* develops around 2 so-called keys [24]. On the one hand, One Health looks at monitoring and assessing environmental hazard interactions and their impacts on health and biodiversity [25]. In contrast, Digital Health stands as the mature deployment of currently available technology to improve individuals’ health and care [26]. The framework also includes 3 perspectives, 5 dimensions, and a technological ring.

As ODH proposes a new holistic, data-driven approach encompassing human, animal, and environmental health and welfare, a solid common ground with this working scenario emerges. Table 1 adjusts the common aspects of smart cities and digital public health using the ODH framework.

Health professionals traditionally define an intervention as the whole process, starting by defining a protocol; implementing it; systematically evaluating its effects; and then using the results to define, for example, a new therapeutic strategy or a new policy. According to the Digital Health perspective, we refer to *ODH Intervention* as the use of digital and mobile technologies for specific initiatives addressing human, animal, and environmental system needs. In other words, an ODH intervention is a real-world implementation of the theoretical ODH framework, which relies on Findable, Accessible, Interoperable, and Reusable (FAIR) data [27-29]. In this regard, we recognize an appropriate alignment with the *Classification of Digital Health Interventions v1.0* proposed by the World Health Organization (WHO), which deals with the principles of implementing and delivering formal or informal care using electronic health services [10,30].

**Table 1:** Relationships between smart cities, digital public health, and One Digital Health (ODH).

Topics in common between (digital) public health and smart cities		ODH dimensions				
		Citizen engagement	Education	Human and veterinary health care	Industry 4.0	Surrounding environment
**Surveillance**						
	Accidents and emergencies				✓	
	Environmental conditions					✓
	Electromagnetic radiation					✓
	Health condition of older adults		✓	✓		
	Emotions	✓				
	Epidemics			✓		✓
	Fitness activities	✓	✓	✓		
	Food quality			✓	✓	✓
Healthy lifestyle promotion		✓	✓	✓		

Our main objective was to show and discuss how the ODH framework—combined with A&EI—can dynamically support the comprehensive monitoring and analysis of the daily life events of humans and animals in technologically integrated environments such as a smart home and a smart city.

The paper is structured as follows. After the introduction, we present and analyze a timely use case from a smart healthy city context [31]. We then describe the planning of an ODH intervention to deal with the use case features. Therefore, we remodel and update the original use case as an actual outcome of an ODH intervention. In the end, we introduce a part of the generic evaluation process of an ODH intervention and discuss its implementation potential. We conclude by pointing out the strengths and limitations of this use case, such as future developments of the ODH framework jointly with A&EI.

## Use Case

### Overview

A smart and healthy city well acknowledges several core ODH challenges. Our use case describes the sequence of steps needed to set up an ODH intervention around a user-centered demand and measure several parameters continuously, comprehensively, and reliably for effective multipurpose and integrative welfare monitoring. In this use case, we deal with human and animal health and welfare monitoring, continuously measuring physiological and behavioral parameters during their different activities—alone or together.

**Figure 1:**
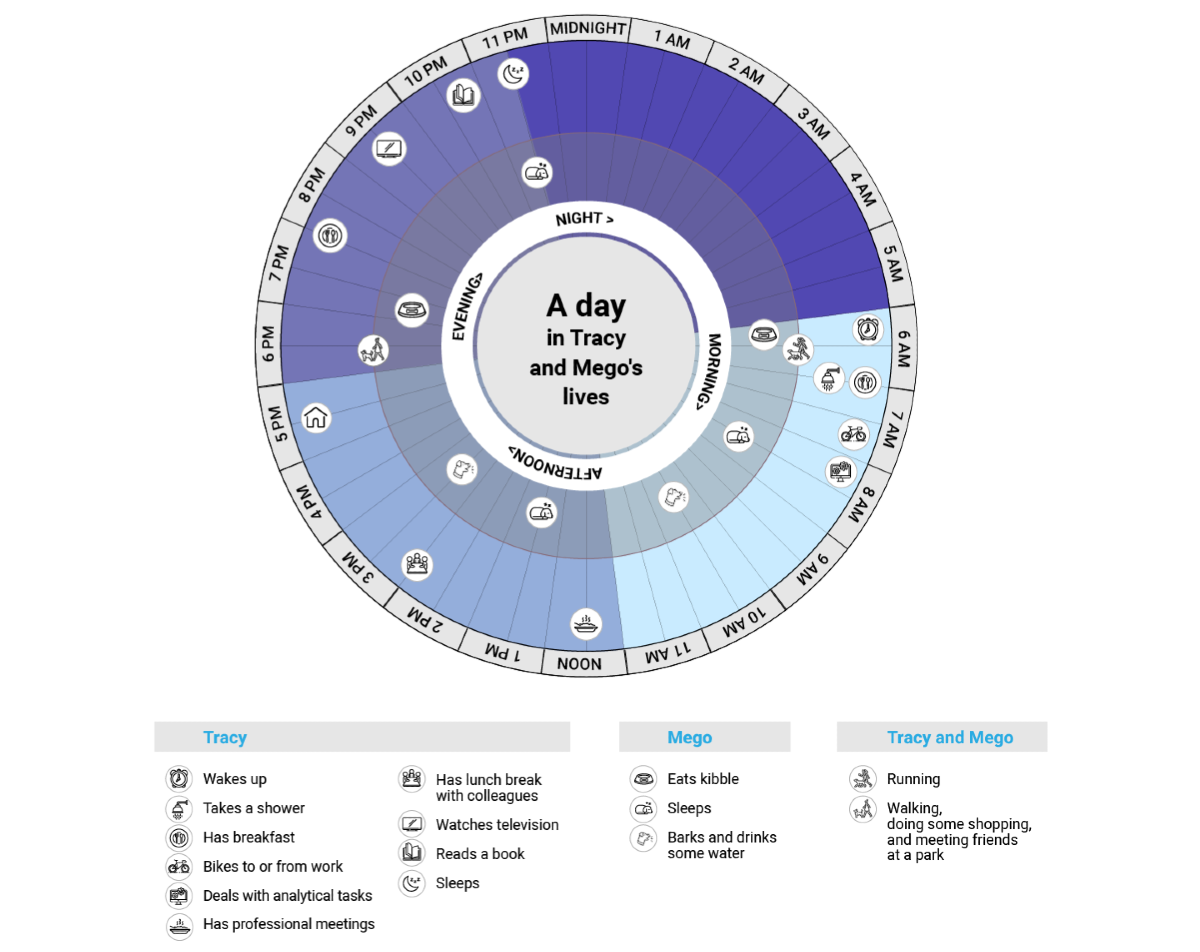
A day in Tracy and Mego’s lives.

Tracy is a healthy single woman aged 27 years who shares her apartment in a metropolitan area with her dog Mego. Tracy is a junior finance analyst (ie, a tertiary position with responsibilities that might cause stress). During working weekdays, Tracy follows some typical activities of daily living. Mego is a healthy midsized male Shiba Inu dog aged 5 years. Mego lives with his owner Tracy. Most of Mego’s time is spent at home (Figure 1).

### A Regular Day in Tracy and Mego’s Lives

At 6 AM, Tracy usually wakes up and checks that Mego’s smart pet feeder delivered kibble and water. She then goes for a 30-minute run with Mego. Returning at 6:50 AM, Tracy takes a shower and has breakfast while briefly checking emails, social media, and the news. At 7:30 AM, she goes to the office by car or bicycle depending on the weather. Tracy works from 8 AM to 5:30 PM. She starts by planning and reviewing professional emails and then tackles high-priority tasks before handling analytical tasks. She takes a 1-hour lunch break with colleagues approximately at noon. The afternoon is organized around meetings and administrative tasks.

To return home, if Tracy used the bicycle on her way to work and there are issues on the way back, she may choose to take it on the bus or train. During this time, Mego, similar to other dogs, rests for a large part of the day and occasionally wakes up and barks to react to surrounding noises, plays with his toys, or drinks some water [13].

At around 6 PM, Mego warmly welcomes Tracy as he is excited to go out. If the weather, unexpected events, and mood conditions allow, they spend approximately 1 hour on a walk. During this time, Tracy does some shopping and meets friends in a park [32]. By 7:15 PM, they return home, and Mego receives his evening food ration. Tracy cooks dinner, watches television, reads a book, and prepares her plans for the next day. At 11:30 PM, Tracy goes to sleep.

Figure 1 shows a 24-slice clock in which the main activities of Tracy and Mego are reported depending on the moment of the day. In particular, an inner circle is recognizable that reports Mego’s activities (eg, sleeping or eating kibble). An external circle features Tracy’s activities (eg, moving to or from work or reading a book). Activities involving both of them are instead reported on the partition line between the aforementioned circles.

### Some Important Spatial-Temporal Facts in Tracy and Mego’s Daily Lives

#### Activity

Tracy spends most of her time indoors at home, her working place, shopping centers, restaurants, and the gym. Outdoor activities include running, working, hanging out with friends in parks and other open spaces, and using public transportation [33-35].

Tracy uses different means of transportation, such as walking, bicycle, car, and public vehicles. From home to work and back, she takes approximately 1 hour of biking—a considerable physical activity. Tracy’s smartwatch informs her of caloric consumption, pulse, and oxygen saturation. The last physiological measurements are regularly stored in the cloud, allowing Tracy to follow and share her physical activities [8]. Mego’s sensor-endowed collar (an IoT device) connects to Tracy’s smartwatch so that she can also monitor his activity and vitals. Most of the day, Mego rests (60% of the time; 14.5 hours a day). However, he has moderate activity (30% of the time; 7 hours a day), such as reacting to environmental stimuli (eg, welcoming Tracy when she returns home or barking at external noises). Mego has an intense physical activity (10% of the time; 2.5 hours a day) when he goes out for a walk or run with Tracy, plays with other dogs in the park, or is at home with his toys either alone or with Tracy [13].

#### Sleep and Rest

When going to bed, Tracy takes off her smartwatch for charging. This is also to prevent this device from disrupting her sleep quality. Tracy turns on her smart home speakers for relaxing music. She also bought a pair of blue light–blocking glasses to guarantee high-quality naps [36]. Mego rests most of the time [13] on a dog bed in Tracy’s room.

#### Nutrition

Tracy has breakfast and dinner at home. Her smart fridge provides potential recipes with the available products [37,38]. She eats catering food at work, such as crudités, cooked legumes, and other protein sources. The cashier’s receipt comprises all the related nutritional intakes [39-41].

Following the recommendation of the veterinary physician, Mego eats kibble. Tracy owns a sensor-endowed feeding station for effective feeding control and regulation [42]. In case of a product recall, Tracy receives a message on her mobile phone explaining the relevant “what to do’s” [43].

### A Particular Day in Tracy’s Life—Example of a Health-Related Disruptive Event

One day, during a busier and more stressful period than regular at work, Tracy took her bicycle to work, but because of the weather, she took the train back home. Afterward, when walking in the park with Mego, Tracy suddenly collapsed. Unfortunately, she had not met her friends yet, nor had anyone happened to be around. Mego noticed the problem and started barking. After a while, some people came along, and finally, the ambulance arrived. Tracy stayed a few days in the hospital and another week at home to recover. During this time, Tracy’s friends had been taking care of Mego, hosting him and trying to preserve his routine to reduce the negative impact of Tracy’s absence (ie, during hospitalization) or unavailability to take care of him on her own (ie, during recovery).

## Methods

### Planning an ODH Intervention

An ODH intervention can be seen as the implementation of a set of digital functionalities, or digitalities [24], designed and deployed to (1) support specific initiatives that address human, animal, and environmental system needs and challenges; (2) assess and study these systems’ expected and unexpected outcomes and effects and collect related data; and (3) select timely metrics for the outcomes of multicriteria decision analyses.

An ODH intervention is implemented to (1) *address One Health–related challenges*; (2) *achieve One Health–related* important and strategic *outcomes* for clinical follow-up and practice, such as for technology improvements needed; and (3) *achieve* FAIR uses of *digital technologies* [30,44].

In our case, this translates to (1) *a challenge* aiming to enhance aspects of healthy lifestyle promotion and surveillance; (2) *an outcome* consisting of performing effective monitoring of human and animal welfare within the context of a smart city, where health is a pivotal component; and (3) sensors for monitoring as *digital technologies* through which the intervention is implemented.

Effective monitoring of humans and animals in a smart environment must cover all relevant locations (indoors and outdoors) and define the critical parameters of health care and QoL [45]. Table 2 reports the 9 major types of interactions between the intervention recipients considering the extant strict interconnectedness between them [46]. Each cell reports instances of how one actor (row) positively or negatively affects another (column).

**Table 2:** Examples of interactions between the 3 One Health components.

	Human	Animal	Environment
Human	Face to face [6] Interactive (technology-based; eg, via social media) [8,47]	Food, habitat provision, emotional bond, and health and well-being control [48] Population follow-up and birth control [49,50] Environment destruction [51]	Compliance and sustainability for a technological environment at home, on a bicycle, in the steering wheel of a car, in the fridge handle, and more [52] Urbanization [53-55] Climate change, pollution, and regulation [51]
Animal	Emotional and physical bond; health, well-being, and safety feelings (eg, dog) [56] Disease vectors (zoonoses) [57]	Food chain [58,59] Natural regulation of populations [60]	Soil fertilization [61,62] Natural hazard (invasive species) [63-66]
Environment	Food chain [58,59] Natural hazards and disasters [67] Space for well-being development [68-70]	Availability of survival resources (food and shelter) [71] Chemicals’ influence on animal reproduction [72]	Wildfire impacts on slope stability triggering in mountain areas [73] Natural hazards and disasters [67]

Therefore, all the collected physiological, behavioral, and environmental data must (1) comprise indoor and outdoor locations where subjects are active and in contact [74,75], (2) be continuously time stamped, and (3) be shareable in a FAIR way [27].

### Adverse Health Events

The ODH framework delivers a variety of observations for continuous health monitoring. It aims at analyzing health data on environmental, behavioral, physiological, and psychological domains, which is in line with the WHO QoL definition [76]. Continuous health monitoring allows continuous data analytics and even subtle trends to become recognizable at the early stages. This then opens many options for preventive medicine, hindering adverse health events (AHEs) [77]. However, AHEs will still occur but maybe not as frequently as without continuous monitoring. In such an event, the measurements taken from the subject are helpful. As of today, smart homes, smart cars, smart wearables, and smart clothes send out emergency calls, but so far, useful information—although available at the site—is not transferred because of lacking protocols and communication standards. This is addressed by A&EI [23]—in an emergency, whether an accident or a health-related adverse event, every second in fact counts as it carries data correlated with the individual involved. In a temporal-spatial continuous monitoring supported by the dynamic point of perception, the subject’s associated data are distributed in each measuring smart environment and device. Upon the occurrence of an event, such isolated data silos have to concatenate to build the complete informative understanding of the latest health status of the subject. The International Standard Accident Number (ISAN) takes over the role, aiming at (1) standardizing an event by associating a unique number (token) composed of time and location of the event and ID number, (2) automatically collecting the corresponding isolated data slices of the individual from the alerting system (ie, smart environments and wearables), (3) automatically generating the alert and transferring it to the responding system (ie, emergency service), and (4) simultaneously transferring the vital and nonvital data to the curing system (ie, hospital) for informing the medical personnel before the subject is delivered to the hospital [78,79].

### ODH Intervention Steps

#### Overview

We deconstruct the ODH intervention into single independent events and steps (globally called “activities”) as items to be organized in tabular form, called the ODH intervention table (see Multimedia Appendix 1). For each activity, a number of fields must be identified, which relate to, for example, ODH domains and dimensions considered, digitalities involved, and their eventual mutual linkages (FAIRness levels).

#### Activity Identification

Each activity is assigned to a single row. The field “Activity UID” indicates the unique numerical identifier of the activity within the ODH intervention. The “Activity name” describes the single ODH intervention step. If the activity is broken down into subtasks, each one of them will be evaluated individually (ie, as a single row).

#### ODH Dimension Scores

An ODH dimension score assesses the dimensions of the ODH framework that directly relate to the activity. Each dimension obtains a specific importance value (increasing integer from 1 to 5) that indicates its connection rate to the corresponding activity of the intervention. The dimensions are reported as follows: C (*citizen engagement*), E (*education*), H (*human and veterinary health care*), I (*health care Industry 4.0* as health care services and technologies involving, eg, robots, 3D printing, cutting-edge ITs, and artificial intelligence [24,80]), and S (*surrounding environment*).

#### Main Digitality Domain

The field refers to 1 of the 3 areas of digital functionalities (humanities, animalities, and environmentalities) encompassed by the technology ring within the ODH steering wheel [24]. This field is divided into 2 subfields.

The first one, named “*Speciality*,” reports the digitality explicitly deployed in the corresponding activity. Each speciality is assigned to a single row. This means that each single activity is operationalized by one or more specialities and is characterized by the marking letter of the domain it refers to: H (human), A (animal), or S (surrounding environment). Accordingly, H(Si) denotes the *i*th speciality that operationalizes the general digital functionality from the human domain, which delivers the corresponding activity.

The second subfield, named “*Technology*,” refers instead to one or more technological solutions through which the speciality is deployed. Each technology is assigned a single row as well.

#### FAIR Data and Data FAIRness

Assessing an ODH intervention’s FAIRness is a basic requirement to correctly process the entire management cycle and stewardship of the data collected and shared during the intervention itself. Thus, the design and deployment steps of an ODH intervention need for its data to be FAIR. In this context, it means that the data must be (1) “Findable” by allowing for their discovery and sharing continuously between different monitoring, analyzing, and alerting systems; (2) “Accessible” by allowing the relevant and approved individuals and connected systems (ie, on behalf of accredited organizations) to deal with data and information when, where, and how needed to manage regular and disruptive events; (3) “Interoperable” by involving health-related communication, data exchanges, and processing standard protocols offered in a secured technological framework; and (4) “Reusable” to allow for a systematic, continuous, and intelligent integration of big multidimensional data for primary (eg, real-time clinical and environment monitoring) and secondary (eg, clinical and epidemiological follow-up) uses [29].

More specifically, a FAIR data assessment is conducted for every technology singled out so that an overall score is extracted for the speciality that the technologies refer to [29]. We mark satisfying, good, and total versus inadequate FAIRness of an individual technology with the symbols + or −, respectively.

#### Related Digitality Domain

**Figure 2:**
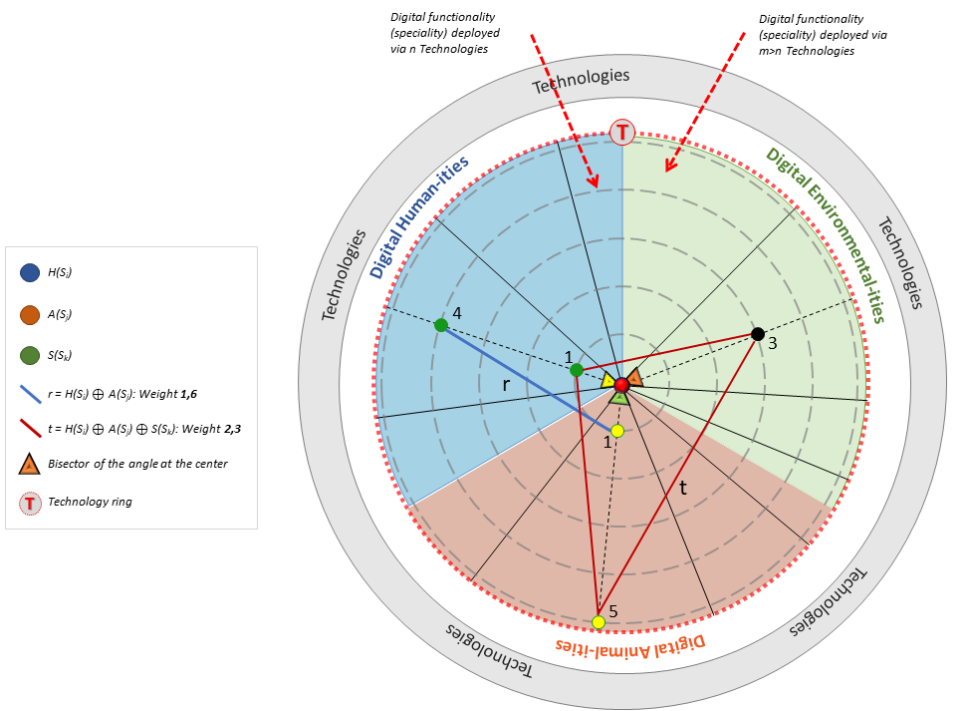
Example of a graphical representation of digitalities involved in a One Digital Health intervention.

This points out 1 of the other 2 areas of the technology ring (Figure 2) involved in the same activity as the main one. Similar to the main digitality domain, we divide related digitalities into the subfields *speciality* and *technology* and assess their level of FAIRness.

#### Data Linkage

In total, 2 digitalities from 2 different domains form a relationship. If 3 digitalities—one from each of the 3 domains—connect with each other, we refer to it as a *triality*, and the field is duplicated. The **“**Data Linkage” field relates to the relationship or triality between the specialities described in the previous subsections. Equation 1 describes an example of a relationship between 2 specialities:

*r_HA_* = *H*(*S_i_*) ⊕ *A*(*S_j_*) **(1)**

where *r_HA_* is the relationship between specialities, *H*(*S_i_*) is the *i*th speciality related to a digital functionality in the human domain, *A*(*S_j_*) is the *j*th speciality related to a digital functionality in the animal domain, and ⊕ denotes a special defined operation similar to addition. It implies a possible confrontation between different specific deployments (in different domains) of the same kind of digital functionality.

In the absence of a relationship between one speciality from one domain and another speciality from another domain, the symbolism of equation 1 becomes as follows:

*r_Hø_* = *H*(*S_i_*) ⊕ ø **(2)**

where ø is the null element (there is no speciality connecting with *S_i_*) and *r_Hø_* is the absence of a relationship (the second member is null). Consequently, we represent a triality as:

*t* = *r_HAS_* = *H*(*S_i_*) ⊕ *A*(*S_j_*) ⊕ *S*(*S_k_*) **(3)**

where *t* = *r_HAS_* is the triality (the specialities relate to all 3 domains) and *S*(*S_k_*) is the *k*th speciality related to a digital functionality in the environment domain.

### Technology Ring

The technology ring serves as a catalyst among all the digital functionalities that the ODH framework relies on [24]. Accordingly, an ODH intervention takes place ideally within it through a series of steps described as follows. Each of the 3 domains is divided into as many circular sectors as digitalities are involved in the ODH intervention (Figure 2).

The width of each circular sector is directly proportional to the number of technologies deployed by the single speciality. Then, the bisector of the angle at the center is identified for each sector and represented by a dashed line. A dot is located along the bisector to represent the rate of involvement of the referring digitality within the ODH intervention.

The position of each dot is the output of the overall assessment of the FAIRness of the digitality considered, which involves all the possible technologies related at once; the lesser the output of the assessment, the farther the dot is from the center, and the higher the associated score (decreasing importance from 1 to 5; integer values) of the digitality.

We define the harmonic average of the associated mentioned scores as the weight of a relationship between 2 digitalities:



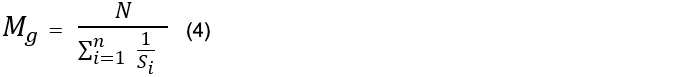



where *M_g_*=*M_g_*(*S_i_*) is the harmonic average, N=2 is the number of digitalities related to each other, and *S_i_* is the score associated with each of the specialities that operationalize the digital functionalities involved in the relationship.

We use a harmonic average as it is based on all observations, gives larger weights to smaller observations, and is thus more robust to strong observations and fluctuations of samples. Moreover, it can cope with variable time factors—in our case, the same speciality deployed via different technologies in different domains and in different time settings.

For a triality, we calculate the *M_g_* value for each of the 3 single relationships and then their arithmetic average. *M_g_* is a function of the scores associated with the 2 related dots. The higher the output of the FAIRness assessment of the digitalities, the closer the corresponding dot is to the center of the technology ring, the lower the ranking of the relationship (decreasing importance from 1 to 5), and the more effective that portion of the ODH intervention is. From a graphical point of view, the dots and relationships shall tend toward the center of the ring. For an optimal ODH intervention, its ODHness (ie, an overall evaluation of how well the ODH intervention is delivered) has to tend to 0 [24].

Eventually, the weight associated with the dimensions involved in the ODH intervention is a corrective factor that improves the scores obtained in the previous steps. Accordingly, the greater the number of dimensions involved in the ODH intervention, the more effective the corrective factor is supposed to be.

### Ethical Considerations

Our research is not an observational study and does not produce or analyze any data taken from humans. Rather, we present a viewpoint and a use case as a potential scenario for an ODH intervention. Accordingly, a review by an ethics committee is not required.

However, planning an ODH intervention must cope with data privacy and security. In our opinion, confidentiality protection is a sensitive issue. In this use case, we do not focus specifically on this aspect. Nevertheless, in a real-world operationalization, it will be critical to have a dedicated data protection framework that allows us to deal when needed with data anonymization and deidentification.

In addition, anyone interested in running (primary use) or using data collected (secondary and tertiary uses) during an ODH intervention in real-world conditions must, according to the relevant local rules, obtain the approval of the relevant research ethics and data protection committees.

## Results

### Use Case (Updated): A Day in Tracy’s Life in a Smart Healthy City

To picture this vision, we flashback to our use case with Tracy and Mego. This time, we tell the same story but set in a smart city using smart devices.

Tracy wakes up while her smart home processes the physiological measurements acquired overnight with IoT devices: a radar sensor on top of the bed monitors the respiratory rate, and the bed is equipped with a capacitive electrocardiograph in the mattress. Tracy also uses ballistocardiography and seismocardiography mounted on the bed frame for cardiorespiratory measurements and sleep assessment. When Tracy opens a door or presses a switch, her body temperature, electrodermal activity (EDA), and photoplethysmography (PPG) are taken, which deliver heart and respiratory rates upon touching the smart door handle or smart switch, respectively. In addition, passive infrared sensors gather activity data for both Tracy and Mego. In the bathroom, the smart toilet analyzes her urine and measures the pH value and density of excrements. In front of the mirror, Tracy’s body temperature is measured by an infrared thermal sensor, whereas the weight scale integrated into records PPG and EDA. When watching television, Tracy’s electrocardiogram (ECG) chair records her heart and respiratory rates.

Before Tracy leaves her apartment, she checks for Mego’s general health status, provided by his smart dog collar, on her app. Upon Tracy’s exit, her smart home transfers the health monitoring to her wearable sensors: Tracy’s smart clothes are equipped with an embedded ECG. During all her outdoor activities, Tracy’s wearable device performs the measurement, and data on the air quality are recorded [17,45].

Today, Tracy uses her car to drive to work. Opening the smart car door activates the measuring system, including body temperature, EDA, and ECG from the steering wheel as well as heart and respiratory rates from image-based PPG computed from the smart car’s indoor camera. It is worth mentioning as well that Tracy’s bicycle is equipped with a smart handlebar that also delivers health data.

### A Particular Day in Tracy’s Life (Updated)

Some days ago, the continuous health monitoring system reported to Tracy that she should consult a physician—the sequence and length of atrial fibrillation periods had slightly increased, a well-known harbinger of stroke. However, because of time conflicts, the physician’s appointment was still outstanding when Tracy collapsed during her evening walk in the park with Mego. Instantaneously, Tracy’s smartwatch detected the AHE and asked Tracy to release the alert return. While the countdown had not yet ended, Mego’s collar reported Mego’s extraordinary excitement to Tracy’s smartwatch, which immediately generated the ISAN and sent an emergency alert. Using the ISAN, the rescue team requested access to the smartwatch so as to check previous and ongoing measurements and, therefore, be well prepared when arriving at Tracy’s location. This made it possible to perform the right intervention at the earliest point in time. Tracy recovered soon without staying in the hospital overnight.

### Analyzing the ODH Intervention

Multimedia Appendix 1 showcases examples of “how-to” analysis of several “activities” (2 in regular time and 1 during a health-related disruptive event) as part of a wider ODH intervention. For each one of them, the rates of involvement of the ODH dimensions are scored, and the relevant technical fields are evaluated in light of the FAIR principles prism. Eventually, for each activity, the data linkage formula is also reported.

The first example relates to the monitoring of Tracy’s respiratory rate via a radar sensor on top of her bed. From the A&EI viewpoint, this activity allows for the detection of respiratory abnormalities to be reported, if needed, in real time to an emergency medical service or be collected for future investigation.

The second example relates to the possibility for Tracy to check on the activity of the wearable devices she and Mego use. This may allow Tracy to know that, “in case of emergency” (eg, fever and muscular pain detected in Mego [before] and Tracy [after] may be signs of a zoonotic phenomenon, such as a bacterial infection known as leptospirosis), the system will be able to send relevant data from each of them to their own medical records [81].

In both cases, the industrial- and health care–related dimensions of the ODH framework appear to be highly involved. The surrounding environment is only slightly affected, whereas no notable involvement emerges for the human-related dimensions.

The last example involves all the ODH dimensions. In this case, using a smartwatch to send an alert message reflects some kind of citizen engagement (C) in sharing data with health care providers (M) that need to be trained (E) to use advanced technologies based on industrial standards (I) that can be used in different environments (S).

## Discussion

### Overview

In this viewpoint and use case, our main objective was to show and discuss how the ODH framework will support 24/7/365 technology-based health and environment monitoring to provide the right answer to the right event (related to a human, animal, or place) in the right place at the right time and with the right means. The central challenge in applying ODH in real-world conditions is the integration of digital development and technologies into 1 health concept. This comes with their use to achieve One Health goals rather than redefining and reconceptualizing One Health in the face of technological advancements [10,11,24,29]. In this regard, planning an ODH intervention is critical for taking forward the integration of the 3 main One Health domains (human, animal, and ecosystem) in light of their digital and computational components. The analyzed use case emerges from the combination of smart cities and digital public health. It suggests an around-the-clock scenario of sensor-based welfare monitoring for a human and a pet in a smart environment context—a large part of Tracy and Mego’s lives involves technological measuring and monitoring systems related to IoMT or IoAHT [8,12]. To enhance the understanding of the impact of an ODH intervention and the subsequent assessment of its ODHness, we proposed a way to analyze human and animal activities in different environments by quantifying their relationships with the ODH framework features [24] and their links to technical fields by considering the assessment of the FAIR guiding principles for the 3 areas identified and included within the ODH technology ring [29,76].

### Principal Findings

In the use case supporting our viewpoint, as in real life, the large amount of data generated by IoT sensors and technologies allows for an effective analysis of behaviors, habits, pattern extraction, and medical conditions of different types of subjects in the mid- and long term. In addition to the initial aim of health condition prediction, prevention, and early alert perspective—usually reported in the existing literature for single contexts such as home [82], hospital [83], or even veterinary epidemiology [84] and that ODH already addresses in a unified way—a growing importance has arisen for disruptive events and punctual abnormalities.

For Tracy, this could be related, for instance, to a medical emergency, such as dyspnea or cardiac arrest during sleep or a physical activity, detected by physiological sensors. It can be, as another example, a bicycle accident detected by physiological sensors, accelerometers, and automatic alerts to emergency services. An additional example relates to contact tracing via an app for highly contagious diseases such as COVID-19, which can alert Tracy and support her in making timely decisions according to the public health authorities’ recommendations.

For Mego, a disruptive event can be a veterinary emergency in case of an accident, as seen for Tracy, or a change in the hydration frequency that can suggest a food-based intoxication.

Moreover, a disruptive and dangerous event can involve both Tracy and Mego, such as a fire, with Tracy’s physiological parameters dramatically changing within a couple of minutes overnight and Mego’s similarly agitated behavior.

Such aspects of emergency management demand a continuous follow-up of humans’, animals’, and surrounding environments’ health care in a P5 medicine approach [85-88] that combines (1) prediction, acquiring data and building relevant models supporting emergency and disaster preparedness [8]; (2) prevention, detecting (weak) signals of abnormalities and treating them before strong deviations (eg, preventive confinement to avoid an epidemic); (3) participation, involving the handling of human, animal, or environmental issues (eg, engaging in vaccination campaigns [89-91]); (4) personalization, proposing the use of the adapted solutions to a detected issue (a drug-based treatment for a human or an animal or the development of a repopulation program for a hurt vegetable ecosystem); and (5) precision, delivering the right intervention at the right time (as much as possible in real time) by the right individual on the right ecosystem components to get to, for example, efficient and dedicated evacuation plans [92,93].

In addition, a specific interpretation is also delivered for what concerns (1) citizen engagement and education aimed at information development and large-scale information gathering (so-called citizen science [94]) for first aid, basic health-related technologies, and sustainability using, for example, mobile apps to increase their penetration rate in the grand public and (2) interoperability from an industrial perspective to facilitate the interconnection and communication between the different IoT systems and alert systems to enhance end-to-end accident and disaster management [78,79].

As shown, singling out the activities that the ODH intervention in a “smart city meets public health” scenario would be made up of and working the technology ring out accordingly (also) to define its ODHness aimed to commence the dialogue with topics from different perspectives dealing with health communication (eg, health promotion based on engagement and education [6,95]) and surveillance (eg, monitoring health conditions and physical activity, detecting and preparing the health care system for disruptive and long-term events, and behavior or environmental changes) [9].

A&EI discipline showed its contributions to the use case regarding the short-term and abrupt event and abnormality detection as well as long-term prediction and prevention. The use-case analysis indicated that A&EI aims to (1) turn smart private spaces into diagnostic spaces unobtrusively (eg, home and car) [96], (2) build continuous measurement and monitoring via dynamic points of perception, and (3) achieve interconnectivity and communication of the means of measurement. The field focuses on the data linkage and interconnectivity of medical and nonmedical sensors and devices in smart environments to construct and support the onboard and distributed data processing and analysis in hierarchical levels of abstracts [12]. The integration of ODH and A&EI in this use case contributes to the development of educational projects and programs, allowing health, environment, engineering, design, and business students and trainees to develop their creative and critical abilities by proposing new concepts and systems [42,95,97]. These education projects could be used in future smart cities wherein One Health and disaster (ie, accidents and emergencies) prevention and preparedness will be daily life pillars [98,99]. This aspect fits with the United Nations 2030 Agenda for Sustainable Development, which points out that young people should be educated in such a way that smart health monitoring is not only for unhealthy humans and older adults [100,101]. For example, analyzing walking steps is a default functionality of mobile devices and is used by young and healthy people to measure their physical activity in real time. Moreover, from an A&EI perspective, preventing complications of a potential medical emergency is something that smartwatches have yet to achieve by detecting and alerting their owners of the first signs of a cardiac event [102].

What emerges is that the very act of planning an ODH intervention placed particular emphasis on aspects of emergency management, which in this context refers directly back to A&EI. This is actually a way to deal with the same dimensions as ODH. Therefore, it is plausible to say that the ODH framework can be enriched with a new layer-like cross-sectional element so that an infinitely recurring loop is created between the 2 models.

In summary, ODH and A&EI as a whole contribute to enhancing (1) the overall QoL of the smart city inhabitants (both humans and animals); (2) the public (digital) health policies and processes frame, such as the entire smart city ecosystem development and management, that is, architecture (eg, accessibility for people with disabilities and reduction of energy consumption) and urbanization (eg, communication systems, transportation networks, malls, education, and health care center locations); and (3) the communication between the different health care system actors involved, such as clinicians, engineers, regulators, and administrators and, more generally, the 3 domains that the ODH framework is made of. The existing Medical Informatics and Digital Health Multilingual Ontology could be expanded and adapted to this end [103,104].

### Viewpoint Constraints

A correct development of the “smart city meets digital public health” field is currently still hindered by a number of factors, as briefly discussed in the following sections.

#### Governance

The control and governance of environmental sustainability can be best approached by assessing ecosystem services that are capable of quantifying and valuing all the goods and services that are generated within the ecosystems themselves. Recently, such networks have been increasingly endowed with digital technologies such as (1) environmental management and monitoring information systems; (2) automated and scalable approaches for collecting, digitalizing, and assembling geocoded big data; and (3) information-fusion algorithms and artificial intelligence that use multiple data streams and clinical decision support algorithms that integrate population-based, public health–focused perspectives into outbreak detection–focused management systems [45,105,106].

#### Data Security and Privacy

However, it is necessary to consider that using electronic and computational systems to collect, store, and analyze data and react and deliver an appropriate response induces at least 2 challenges. Dealing with health care customer data requires dealing with their security and privacy from an ethical viewpoint to ensure patient rights [107] and prevent data theft that can induce, for example, health-related device hijacking with dramatic consequences [108]. Moreover, the health-related data acquisition and monitoring systems used in smart cities demand seamless and secured bidirectional communication, supporting high-speed, large-bandwidth, and low-latency internet infrastructures materialized by the fifth and sixth generation of wireless network technology (respectively known as 5G and 6G) and future versions. Moreover, the use of cloud-based storage and computational resources is essential to allow (near) real-time data collection and analysis.

#### Sustainability

Accordingly, from a sustainability perspective, as smart cities are electric power–based and digital green, digital health green infrastructures must be developed to reduce the environmental fingerprint [109,110].

Therefore, from an industrial perspective, reducing power consumption, electronic waste, and biodegradable health care–related materials is a global challenge [111,112].

#### Interoperability

Furthermore, in smart cities, the heterogeneity of IoT devices, and specifically those related to the health care industry, must benefit from improved interoperability standards and procedures and device compatibility. This last point must critically affect the health service providers’ and customers’ acceptance and use of mobile and ubiquitous technologies [113].

#### Health Communication and Disparity

Nevertheless, a limitation of having a generalized use of IoT devices or, more globally, a healthy smart city lies on health communication and disparity. Indeed, using IoT may be challenging for different groups such as older adults as well as individuals with communication disabilities or limited abilities. Accordingly, even though IoT is taking health care to the next stage worldwide, it could paradoxically exclude some people who need it [6,114]. Therefore, a similar threat may affect the veterinary sector, whose development rate is still quite lagging behind that of human medicine.

#### Ethical and Cultural Limitations

In addition, collecting continuous data on anyone and anything may entail ethical and cultural limitations both related to privacy. In a smart city, data collection and processing are performed such that the customers of health care services are not (fully) aware of the sharing of personal data [115]. Moreover, developing a smart city wherein health monitoring is a pillar requires a high technology acceptance level when we consider that technologies are everywhere. Just to mention another critical sector such as transportation safety, the development of IoT-based vehicle accident detection is important, but privacy limitations exist regarding the fact that any driving event can be recorded even if it is not related to an accident [116,117].

In the use case presented, the use of the different activity trackers points out that the technology is accepted by our persona (Tracy), yet it is an open question whether she was fully aware of “giving up” part of her privacy rights [4,118].

### Limitations of the Study

Some limitations of this work arise from the way the use case for the ODH intervention was determined. First, the “Animal” domain was only represented by a pet. The presence or role of other kinds of animals—such as nonconventional pets (eg, rabbits and reptiles), herds (eg, cows and goats), or wildlife (eg, wolves and boars)—was not addressed. In addition, the “Environment” domain was not properly included in the use case, whereas in a smart and healthy city, the management of environmental resources, made itself smart via IoT and information and communications technologies, is a critical element toward actual e-governance policy planning [119].

Furthermore, a major focus was on the general category of IoT that, although an all-encompassing type of technology, is currently dealing with peculiar declinations for what concerns the general (human) health care sector with IoMT and, in more recent times, animals’ health and well-being status with IoAHT [120].

Another findable limitation is that the proposed use case only describes a single day in Tracy and Mego’s lives. Although Tracy’s working days are characterized by many routine activities, the analysis of a longer period would have likely involved many more things to be discussed.

### Future Perspectives

Tracy’s daily occupations and activities presented in this fictional use case are applicable to a large part of the population living in similar contexts.

One of the potential and expected implications of ODH interventions lies on supporting digital health literacy, from children to older adults, to engage with personal health, public health, and environmental monitoring systems to increase awareness. Improvement in this aspect is likely to lead to better health outcomes and a more proactive approach to medical practice, thus reducing both the digital divide and health inequalities [6,121].

On the basis of these assumptions, the development of policies regarding health data management and sharing for secondary use [122-126] is expected to be facilitated and perhaps automated. A timely and FAIR deployment of ODH- and A&EI-based data collection modalities would lead to the establishment of more comprehensive guidelines and decision support systems for what concerns the many (interrelated) features of health care and to a better understanding of user expectations.

The combination of health literacy, human expectations, and data collection—or, rather, the logical flow through these points—yields scenarios where information is stored in electronic health records in a timely manner and available in health care management organizations’ repositories and allows for the implementation of personalized medicine processes considering environmental measurements and human [127] or animal [120,128,129] behaviors. In a broader, holistic view, it looks at a proactive medical practice that no longer addresses patients but humans outside the human-centered health care systems whose improved awareness even steps beyond the concept of “empowerment” [130,131]. This means that electronic health record data shall be processed with other data such as lifestyle habit data, for example, patient-generated health data (ie, not reported to health care professionals) and environmental data (eg, pet owning [42] and hobbies). An integrative understanding of the human way of life and human needs and expectations will help efficiently and effectively enhance health communication–targeted campaigns to improve disease prevention, detection, and follow-up at a large scale [6].

### Conclusions

Our vision for the future sees smart cities as an emerging paradigm involving a large set of technologies (eg, IoT and, thus, digital health, telehealth, mobile health, and web-based social networks of patients and caregivers) and behavior-changing tools to refine education, engagement, the consumption of food, physical activity, and the use of technology. The main aim is to improve QoL and life expectancy. This has shifted health care focus from treatment after diagnosis to prediction and prevention and from a health care professional–centered (so-called paternalistic [132]) approach to a health care service user–centered follow-up management. Thus, health care is no longer limited to the walls of the health care centers (eg, clinics and hospitals) and the “homespitals” (also known as hospital at home) but is ubiquitous and based on continuous, comprehensive, and reliable measurement of several physiological and behavioral parameters. The integration of the ODH and A&EI viewpoints will allow for the reduction of disparities and loss of time in managing disruptive health-related events and for looking at health as a whole, wherein human and animal well-being in a secure and proactive environment. ODH and A&EI are triggers for developing and implementing precision public health, currently defined as imaginary, by dealing with the entire data management process from end to end [11].

## Appendix

Multimedia Appendix 1 Excerpts of the One Digital Health (ODH) intervention table for this use case.

## Abbreviations

A&EI: accident and emergency informatics
AHE: adverse health event
ECG: electrocardiogram
EDA: electrodermal activity
FAIR: Findable, Accessible, Interoperable, and Reusable
IoAHT: Internet of Animal Health Things
IoMT: Internet of Medical Things
IoT: Internet of Things
ISAN: International Standard Accident Number
ODH: One Digital Health
PPG: photoplethysmography
QoL: quality of life
WHO: World Health Organization

## Data Availability

Data sharing is not applicable to this paper as no data sets were generated or analyzed during this study.
