# Supplementary material for: One Digital Health Intervention for Monitoring Human and Animal Welfare in Smart Cities: Viewpoint and Use Case
Source: JMIR Med Inform. 2023 May 19;11:e43871. doi: 10.2196/43871 (PMC10238965; doi:10.2196/43871)
Supplement: Multimedia Appendix 1 [file medinform_v11i1e43871_app1.pdf]

Multimedia Appendix 1: Excerpts of the One Digital Health (ODH) intervention table for this use case.

ID	Moment of the day	Activity	ODH Dimension(s) + scores					Main digitality domain (H[a]/A[b]/S[c])		Related digitality domain (H/A/S)		Data linkage
			C[d]	E[e]	M[f]	I[g]	S[c]	Speciality(ies)	Technology(ies) and FAIRness[h] level	Speciality(ies)	Technology(ies) and FAIRness level	
1	Night until wake-up (11:30 PM-6 AM)	"Respiratory rate is monitored by a radar sensor on top of the bed."	0	0	5	4	2	Biomedical engineering: $H(S_i)$	• IoT[i] (biomedical sensor)<br>*Findable: F1+*<br>*Accessible: A1.2–*<br>• IoT (domotics)<br>*Reusable: R1.2+*<br>• Signal analysis<br>*Findable: F3–*<br>*Interoperable: I2+*<br>• Standards<br>*Interoperable: I1+*	N/A[j]	N/A	$H(S_i) \oplus$ ø
2	All throughout the day when relevant	"Mego's body temperature and general health status are recorded on his wearable device. Tracy can monitor such data via a dedicated app on her smartphone."	0	0	5	4	2	Biomedical engineering: $A(S_i)$	• IoT (biomedical sensor)<br>*Findable: F1+*<br>*Accessible: A1.2–*<br>• IoT (wearables)<br>*Reusable: R1.2+*<br>• Signal analysis<br>*Findable: F3–*<br>*Interoperable: I2+*<br>• Standards:<br>*Interoperable: I1+*	Electrical engineering: $H(S_1)$<br><br>Software engineering: $H(S_2)$	• Electrical engineering:<br>○ *Signal* processing<br>*Findable: F3+*<br>○ *Standards*<br>*Interoperable: I1–*<br>• Software engineering:<br>○ *Coding:*<br>*Interoperable: I1+*<br>○ *Interoperability*<br>*Accessible: A1.1–*<br>*Interoperable: I1+*	$A(S_i) \oplus$ $[H(S_1) + H(S_2)]$

3	Evening (at approximately 6 PM)	"Tracy collapsed in the park. Her smartwatch sent an alert message. Using the ISAN[k], the rescue team requested access to the smartwatch."	2	3	3	5	3	Communication engineering: H(S_i)	• IoT (general)<br>*Findable: F1+*<br>*Accessible: A1.2–*<br>• Information system security<br>*Interoperable: I1+ and I3+*<br>*Reusable: R1.2+ and R1.3+*<br>• Standards<br>*Interoperable: I1+*	N/A	N/A	H(S_i) $\oplus$ ø

[a]H: human domain.

[b]A: animal domain.

[c]S: surrounding environment (domain and dimension).

[d]C: citizen engagement.

[e]E: education.

[f]M: medical (human and veterinary health care).

[g]I: Industry 4.0.

[h]FAIRness: principles of (meta)data Findability, Accessibility, Interoperability, and Reusability.

[i]IoT: Internet of Things.

[j]N/A: not applicable.

[k]ISAN: International Standard Accident Number.
